# Redo coronary bypass grafting for congenital left main coronary atresia: a case report

**DOI:** 10.1186/s13019-017-0588-2

**Published:** 2017-05-15

**Authors:** Shin Yajima, Koichi Toda, Hiroyuki Nishi, Daisuke Yoshioka, Teruya Nakamura, Shigeru Miyagawa, Yasushi Yoshikawa, Satsuki Fukushima, Yoshiki Sawa

**Affiliations:** Department of Cardiovascular Surgery, Osaka University Graduate School of Medicine, 2-15 Yamadaoka, Suita, Osaka 565-0871 Japan

**Keywords:** Left coronary main atresia, Redo surgery, Coronary artery bypass grafting, Congenital heart disease, Bilateral internal thoracic artery, Mitral annuloplasty, Case report

## Abstract

**Background:**

Congenital left main coronary atresia is an extremely rare coronary anomaly. Long-term surgical outcomes and the optimal management strategies for recurrence of ischemia remain uncertain. Herein, we present a case involving successful redo coronary artery bypass grafting for unstable angina 27 years after the initial coronary artery bypass grafting for congenital left main coronary atresia.

**Case presentation:**

A 33-year-old woman was referred to our department with unstable angina. At the age of 6, she had undergone coronary artery bypass grafting of the second diagonal branch using the left internal thoracic artery and the obtuse marginal branch using saphenous vein grafting for left main coronary atresia. Although a coronary angiogram showed a patent left internal thoracic artery graft to the second diagonal branch and a patent saphenous vein graft to the obtuse marginal branch, the left anterior descending artery was not being perfused by the grafts because of a disruption of blood flow to the left anterior descending artery from the left internal thoracic artery. Therefore, we performed a redo coronary artery bypass grafting using the in situ right internal thoracic artery to the first diagonal branch, which was to be connected to the left anterior descending artery, resulting in amelioration of the ischemia of the left anterior wall. The patient was discharged 10 days after the operation and has been in good health for over 3 years without recurrence of chest symptoms.

**Conclusions:**

Coronary revascularization using a saphenous vein and left internal thoracic artery grafts is effective in achieving an adequate blood supply to the distal coronary arteries, and this effect can last for decades. However, careful follow-up is necessary because recurrent myocardial ischemia due to the development of a coronary artery occlusion may occur in adulthood.

## Background

The incidence of coronary artery anomalies has been reported to be 0.6–1.3% in an angiographic series and 0.3% in an autopsy series [1]. Congenital left main coronary atresia (LMCA) is an extremely rare anomaly with only approximately 60 cases being described in the literature, in which the left main coronary ostium ends blindly and the left anterior descending artery (LAD) is perfused via poor collateral flow from the right coronary artery. Although surgical intervention achieves a good short-term result, its impact on the long-term outcomes and indications for a secondary intervention remain uncertain [2]. Herein, we report a case involving successful redo coronary artery bypass grafting (CABG) for unstable angina 27 years after the initial CABG for congenital LMCA.

## Case presentation

**Fig. 1:**
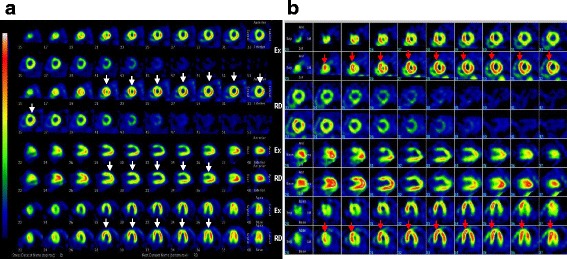
Scintigraphy before and after the redo coronary artery bypass. **a** Exercise stress myocardial perfusion scintigraphy using technetium tetrofosmin reveals an extensive area of ischemia on the anterior *left* ventricular wall before redo coronary artery bypass grafting (*white arrows*). **b** Adenosine triphosphate stress myocardial perfusion scintigraphy using technetium tetrofosmin reveals blood flow recovery in the intraventricular septum after the redo coronary artery bypass grafting (*red arrows*)

**Fig. 2:**
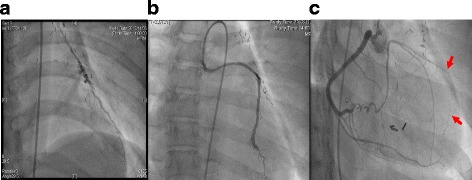
Coronary angiogram before the redo coronary artery bypass grafting. Coronary angiogram before the redo coronary artery bypass grafting shows patent bypass grafts 27 years after the initial coronary artery bypass grafting to the second diagonal branch using the *left* internal thoracic artery (**a**), and to the obtuse marginal branch using a saphenous vein graft (**b**). However, the *left* anterior descending artery (*red arrows*) was supplied only via poor collateral flow from the right coronary artery (**c**)

**Fig. 3:**
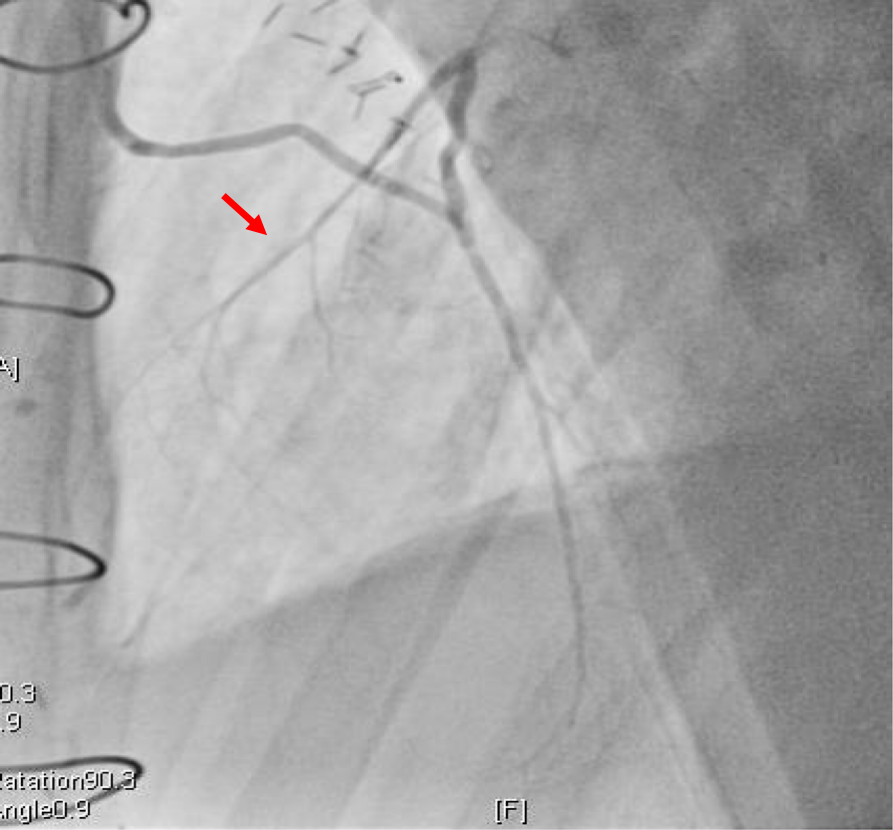
Coronary angiogram after the redo coronary artery bypass grafting. Coronary angiogram after the redo coronary artery bypass grafting shows a patent *right* internal thoracic artery and blood flow communication between the first diagonal branch and the left anterior descending artery (*red arrow*)

A 33-year-old woman was referred to our department with unstable angina. At the age of six, she underwent CABG to the second diagonal branch using the left internal thoracic artery (LITA) and to the obtuse marginal branch using a saphenous vein graft (SVG), as well as mitral annuloplasty for congenital LMCA and moderate mitral regurgitation. After the initial operation, 18 years passed without any signs of angina. However, at the age of 24, she started to experience occasional chest pain on exertion, which had become more frequent by the age of 32. Although her electrocardiogram and echocardiogram showed no abnormal findings, exercise stress myocardial perfusion scintigraphy revealed an extensive ischemic lesion on the left ventricular anterior wall (Fig. 1a). Although a coronary angiogram showed a patent LITA to the second diagonal branch (Fig. 2a) and a patent SVG to the obtuse marginal branch (Fig. 2b), the LAD was not perfused by the LITA, and was mainly supplied by collateral flow from the right coronary artery (Fig. 2c). Multidetector-row computed tomography demonstrated a disruption of blood flow to the LAD from the LITA due to an occlusion of the proximal part of the second diagonal branch. Therefore, to improve the ischemia of the LAD lesion, we performed a redo CABG using the right internal thoracic artery (RITA). In the current case, because the distal LAD was too small to be grafted, and the proximal LAD was deep in the myocardium, we bypassed to the first diagonal branch, which was connected to the LAD. Postoperative coronary angiogram showed that all bypass grafts, including the RITA, were patent and there was blood flow communication between the first diagonal branch and the LAD (Fig. 3). Pharmacologic stress perfusion scintigraphy revealed an improvement in the ischemia, especially in the left intraventricular septum (Fig. 1b). The patient’s symptoms also improved and she was discharged 10 days after surgery. She has been in good health for over 3 years without recurrence of chest symptoms.

## Discussions and conclusions

LMCA is roughly classified into two categories according to age at clinical presentation (pediatric or adult). Pediatric patients are likely to develop clinical presentations such as syncope, decompensated heart failure, ventricular tachycardia, or myocardial infarction earlier than adults because they often have oxygen-consuming comorbidities. Surgical reconstruction is recommended in patients with LMCA and surgical reconstruction has been reported to provide good short-term results [3]. However, the long-term outcomes following surgical revascularization for LMCA remain unknown, especially when vein grafts are used.

In a series of patients with Kawasaki disease who underwent CABG, Kitamura and colleagues reported good long-term patency of the internal thoracic artery regardless of patient age, while long-term SVG patency was significantly reduced in patients younger than 10 years [4]. In the present case, the patient had survived for 27 years after the initial CABG for congenital LMCA with evidence of patent grafts, including the SVG.

For reason why an occlusion occurred proximal to the second diagonal branch, the progression of coronary artery disease was considered to be less likely in our case because she was young and without concomitant arteriosclerotic disease. Although early development of coronary artery stenosis 8 months after reconstruction of the left main trunk with an azygos vein patch has been reported in a patient with LMCA [5], occlusion of the coronary artery CABG to LMCA was not reported in the long-term follow-up. One possible reason was anastomotic technical difficulties with the heels of the anastomoses because of likely small size of coronary arteries in a 6 year old child. As another possible reason, flow competition between an internal thoracic artery graft to the LAD and an SVG to the obtuse marginal branch has been reported as a cause of narrowing of the internal thoracic artery [6]. Thus, we speculated that the blood flow from the SVG anastomosed to the obtuse marginal branch and that the collateral flow from the right coronary artery may have competed with that from the LITA, resulting in occlusion of the proximal part of the second diagonal branch.

The RITA was selected as a graft because internal thoracic artery bypasses have shown superior long-term patency and greater physiological adaptability to various flow patterns [7]. Postoperative myocardial blood flow recovery supports our redo CABG strategy. This case may provide useful information regarding the long-term outcomes of CABG to the LMCA. Coronary revascularization using a SVG and a LITA graft is effective in achieving an adequate blood supply to the distal coronary arteries, which can last for decades (even with a SVG). However, careful follow-up is necessary because recurrent myocardial ischemia due to the development of a coronary artery occlusion may occur in adulthood.

## Abbreviations

CABG: Coronary artery bypass grafting
LAD: Left anterior descending artery
LITA: Left internal thoracic artery
LMCA: Left main coronary atresia
RITA: Right internal thoracic artery
SVG: Saphenous vein graft

## References

[1] Yildiz A, Okcun B, Peker T, Arslan C, Olcay A, Bulent VM (2010). Prevalence of coronary artery anomalies in 12,457 adult patients who underwent coronary angiography. Clin Cardiol.

[2] Tanawuttiwat T, O’Neill BP, Schob AH, Alfonso CE (2013). Left main coronary atresia. J Card Surg.

[3] Musiani A, Cernigliaro C, Sansa M, Maselli D, De Gasperis C (1997). Left main coronary artery atresia: literature review and therapeutical considerations. Eur J Cardiothorac Surg.

[4] Kitamura S, Tsuda E, Kobayashi J, Nakajima H, Yoshikawa Y, Yagihara T (2009). Twenty-five-year outcome of pediatric coronary artery bypass surgery for kawasaki disease. Circulation.

[5] Takeuchi D, Mori Y, Kishi K, Nakajima T, Nakazawa M, Tsurumi Y (2009). Percutaneous transluminal coronary angioplasty for postoperative left coronary artery stenosis following surgical reconstruction of congenital atresia of the left main coronary artery. Circ J.

[6] Kawamura M, Nakajima H, Kobayashi J, Funatsu T, Otsuka Y, Yagihara T (2008). Patency rate of the internal thoracic artery to the left anterior descending artery bypass is reduced by competitive flow from the concomitant saphenous vein graft in the left coronary artery. Eur J Cardiothorac Surg.

[7] Singh RN, Beg RA, Kay EB (1986). Physiological adaptability: the secret of success of the internal mammary artery grafts. Ann Thorac Surg.

